# Supplementary material for: Intersection of rare pathogenic variants from TCGA in the All of Us Research Program v6
Source: HGG Adv. 2025 Jan 11;6(2):100405. doi: 10.1016/j.xhgg.2025.100405 (PMC11830373; doi:10.1016/j.xhgg.2025.100405)
Supplement: Document S1. Figures S1–S3 [file mmc1.pdf]

HGGA, Volume *6*

Supplemental information

Intersection of rare pathogenic variants from TCGA in the All of Us Research Program v6

Blaine A. Bates, Kylee E. Bates, Spencer A. Boris, Colin Wessman, David Stone, Justin Bryan, Mary F. Davis, and Matthew H. Bailey

Figure S1: Anatomical origins of neoplasms. The bar plot displays participant counts that illustrate cancer cases based on self-reported occurrences from surveys, electronic health records (EHRs), or both (according to SNOMED breakdown). Cancer events are categorized by major organ systems. If a participant had multiple cancers, they were counted in each affected organ system.

Figure S2: **Comparison of age distributions and cancer occurrence among broad groups of genetic similarities in *All of Us*. (A)** The age density distribution is presented for each ancestry, with age on the x-axis and density on the y-axis. **(B)** The mean age (x-axis) is plotted against the fraction of individuals exhibiting a cancer phenotype (y-axis) for each ancestry. Error bars represent the 95% confidence interval for the true mean age of each ancestry. **(C)** Time-to-cancer curves are created for each ancestry using the sample colors from panels A and B. Time is displayed in years on the x-axis, while the fraction of *All of Us* samples without a cancer diagnosis is shown on the y-axis. (Below) Two genetic ancestries, African (bottom-left) and European (bottom-right), indicate whether carrying rare predisposition mutations (darker shade) results in earlier cancer onset than non-carriers (lighter shade).

Figure S3: **Comparison of effect sizes between European-like and non-European-like genetic ancestries for selected *BRCA2*-cancer associations from our predisposition variant PheWAS**. Effect size is plotted on the x-axis for seven of the associations that were statistically significant in the European genetic ancestry PheWAS. Error bars show two times the standard error for the effect size.

Table S1: **Summary of TCGA germline variant data in *All of Us*.** Shows genomic position and reference/alternate alleles from TCGA as well as the genomic position and reference/alternate

alleles as specified in *All of Us*. The count of individuals in *All of Us* with a given variant is also given.

Table S2: Details for the results from the cancer-specific predisposition gene association study. Summary statistics are shown for each test that was run.

Table S3: **Details for the significant gene-phenotype associations in the pathogenic variant PheWAS (both European and non-European genetic ancestries).** Summary statistics for each association are given.

Figure S1

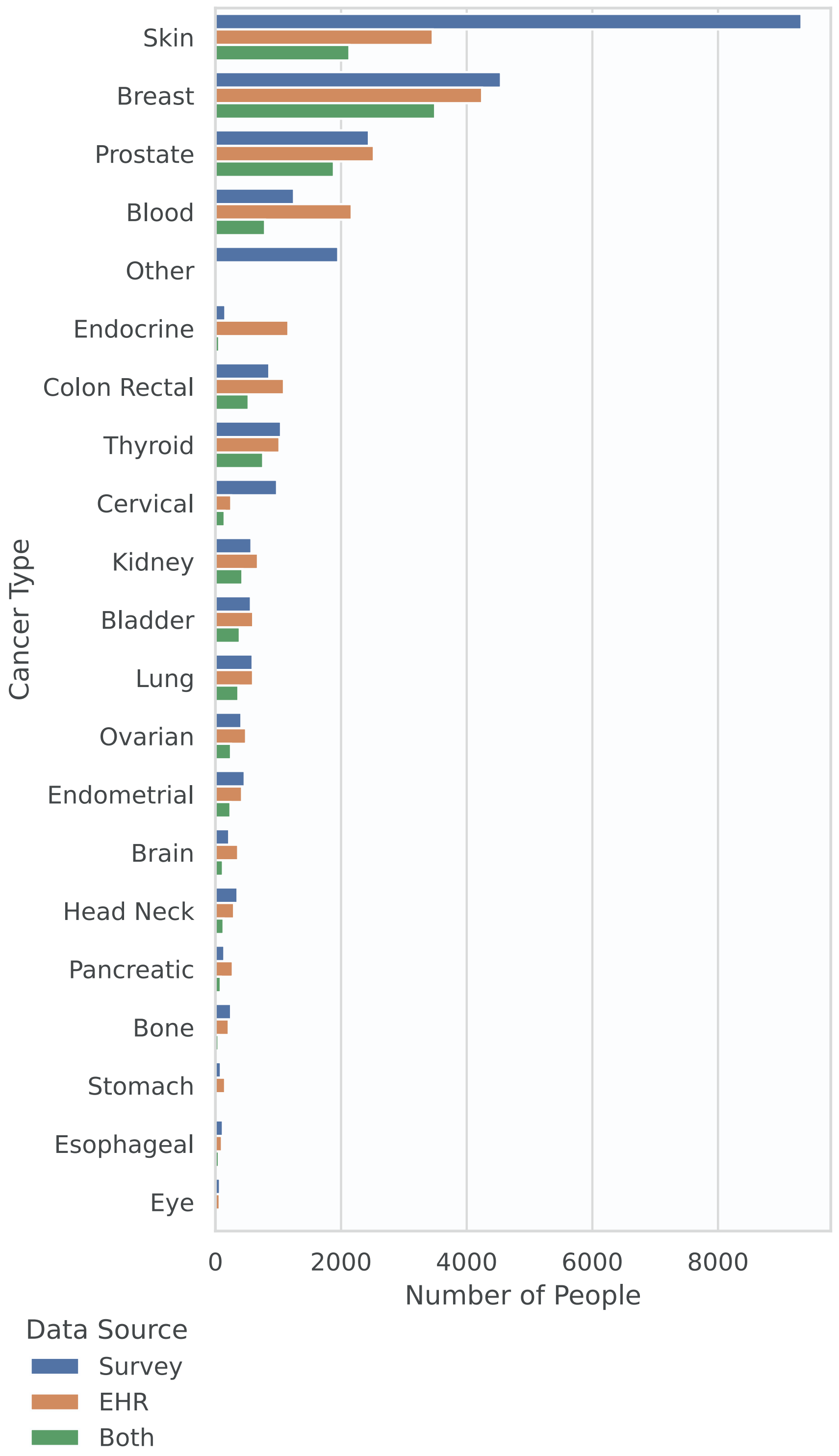

Skin
Breast
Prostate
Blood
Other
Endocrine
Colon Rectal
Thyroid
Cervical
Kidney
Bladder
Lung
Ovarian
Endometrial
Brain
Head Neck
Pancreatic
Bone
Stomach
Esophageal
Eye
Cancer Type
0
2000
4000
6000
8000
Number of People
Data Source
Survey
EHR
Both

Figure S2

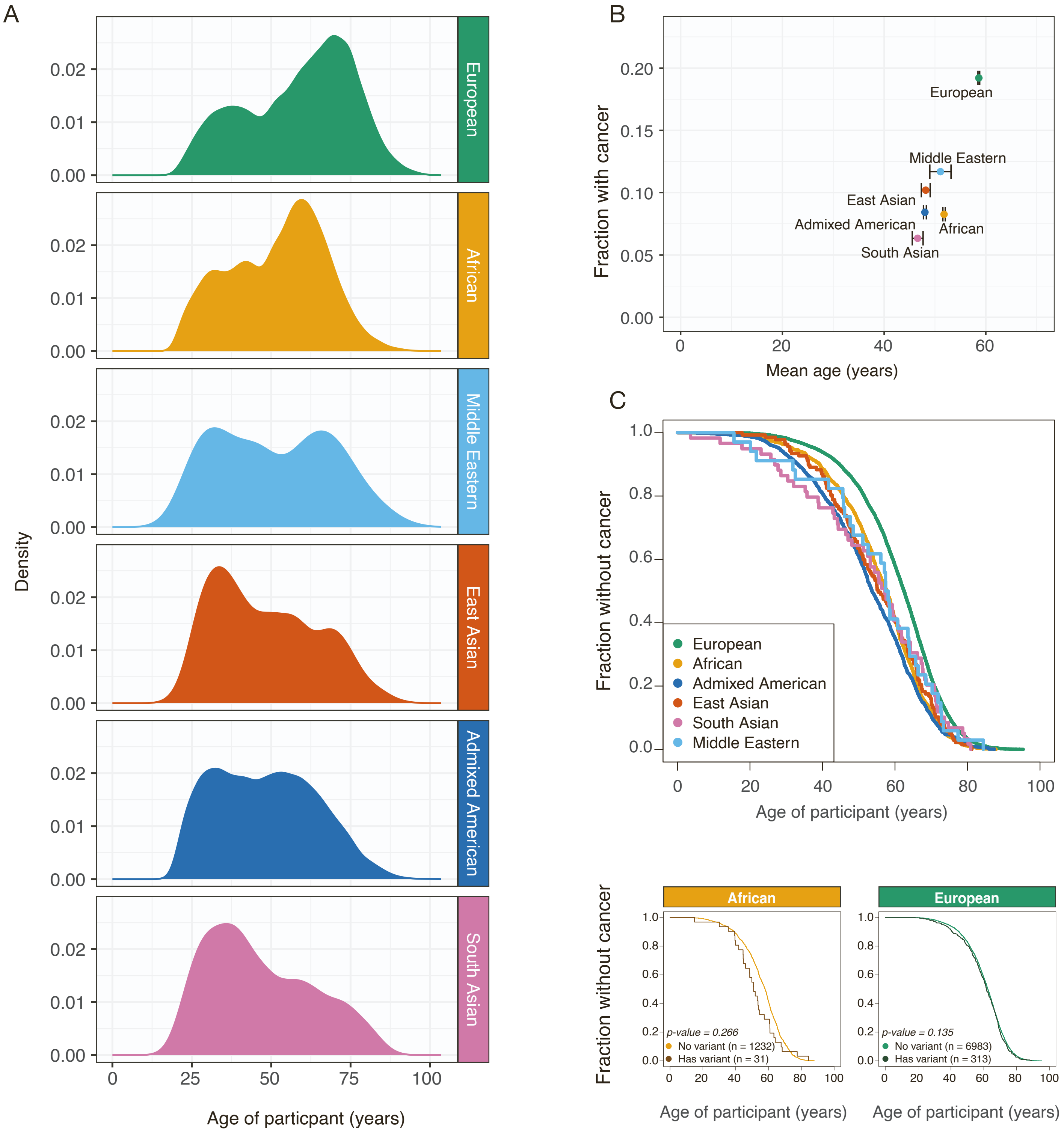
A
Density
European
African
Middle Eastern
East Asian
Admixed American
South Asian
Age of particpant (years)
B
Fraction with cancer
European
Middle Eastern
East Asian
Admixed American
African
South Asian
Mean age (years)
C
Fraction without cancer
European
African
Admixed American
East Asian
South Asian
Middle Eastern
Age of participant (years)
Fraction without cancer
African
European
p-value = 0.266
No variant (n = 1232)
Has variant (n = 31)
p-value = 0.135
No variant (n = 6983)
Has variant (n = 313)
Age of participant (years)
Age of participant (years)

Figure S3

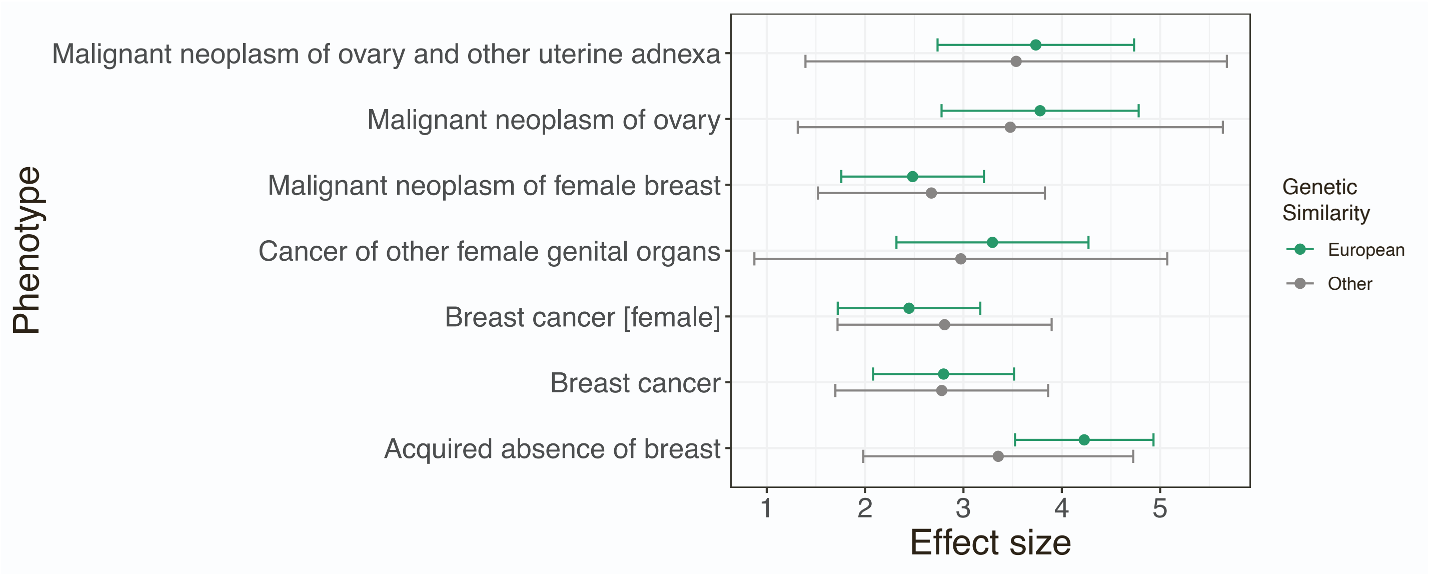

Malignant neoplasm of ovary and other uterine adnexa
Malignant neoplasm of ovary
Malignant neoplasm of female breast
Cancer of other female genital organs
Breast cancer [female]
Breast cancer
Acquired absence of breast
Phenotype
1
2
3
4
5
Effect size
Genetic Similarity
European
Other
