# Supplementary material for: Intersection of rare pathogenic variants from TCGA in the All of Us Research Program v6
Source: HGG Adv. 2025 Jan 11;6(2):100405. doi: 10.1016/j.xhgg.2025.100405 (PMC11830373; doi:10.1016/j.xhgg.2025.100405)
Supplement: Document S2. Article plus supplemental information [file mmc5.pdf]

HGG
Advances

ARTICLE

Intersection of rare pathogenic variants from TCGA in the All of Us Research Program v6

Blaine A. Bates,[1,2] Kylee E. Bates,[3] Spencer A. Boris,[1] Colin Wessman,[1] David Stone,[1] Justin Bryan,[1] Mary F. Davis,[3,4] and Matthew H. Bailey[1,5,6,*]

Summary

Using rare cancer predisposition alleles derived from The Cancer Genome Atlas (TCGA) and high cancer prevalence (14% of participants) in *All of Us* (version 6), we assessed the impact of these rare alleles on cancer occurrence in six broad groups of genetic similarity provided by *All of Us*: African/African American (AFR), Admixed American/Latino (AMR), East Asian (EAS), European (EUR), Middle Eastern (MID), or South Asian (SAS). We observed that germline susceptibility to cancer consistently replicates in EUR-like participants but less so in other participants. We found that *All of Us* participants from the EUR ($p = 1.8 \times 10^{-7}$), AFR ($p = 0.018$), and MID ($p = 0.0083$) genetic similarity groups who carry a rare pathogenic mutation are more likely to have cancer than those without a rare pathogenic mutation. With the advent of combining medical records and genetic mutations, we also performed a phenome-wide association study (PheWAS) to assess the effect of pathogenic variants on additional phenotypes. This analysis again showed several associations between predisposition variants and cancer in EUR-like participants, but fewer in those of the other genetic similarity groups. As *All of Us* grows to 1 million participants, our projections suggest sufficient power (>99%) to detect cancer-associated variants that are common, but limited power (~28%) to detect rare mutations when using the entire cohort. This study provides preliminary insights into genetic predispositions to cancer across a diverse cohort and demonstrates the value of *All of Us* as a resource for cancer research.

Introduction

Multifarious omics research has improved our molecular understanding of cancer. With the advent of next-generation sequencing, the past two decades have produced vast amounts of genotypic and phenotypic data. In particular, The Cancer Genome Atlas (TCGA) curated whole exome variant calls[1] for somatic,[2] RNA-expression,[3] copy number,[4] aneuploidy,[5] and clinical profiles.[6] Additionally, Huang et al. discovered 500+ pathogenic germline variants within the TCGA dataset[7] that likely contribute to cancer. Summatively, this study linked functional consequences to rare predisposition variants that could impact germline testing of cancer. One of the primary limitations of the TCGA cohort is the lack of diversity, reporting that a majority of the cancer types studied were disproportional to races and stages reported in national cancer registries.[8] These discrepancies warrant additional cancer datasets accurately reflecting the genetic diversity in the United States.

To address this issue, and many others concerning human health, the National Institutes of Health has produced the *All of Us* Research Program.[9] This phenomenal program has over 400,000 participants who have agreed to share their electronic health records (EHR), and, as of the version 6 data release (*All of Us* data: allofus.nih.gov), almost 100,000 are paired with whole-genome sequencing. The *All of Us* Research Program plans to provide genome sequencing to 1 million participants and intends to release updated data for researchers' use annually. Participant selection is disease agnostic, and recruitment has focused on underrepresented minorities in the United States, with almost 50% of participants reporting non-White. A preliminary analysis of the insurance billing codes suggests that *All of Us* will be a fruitful dataset for studying cancer. In our study population of 67,598 individuals (see material and methods, Figure 1A), we found 9,780 individuals (14%) with malignant neoplasm records. While 14% is low compared with current estimates of lifetime cancer risk, e.g., >50% according to Ahmad et al.,[10] the disproportionally high fraction of health records linked to participants with malignant neoplasms suggests that the *All of Us* Research Program is a strong dataset to study cancer. Additionally, updates to EHR data for participants will continue to be added to identify patients who develop malignancies in the future.

Here, we provide a preliminary synopsis of cancer genotypes determined by TCGA and phenotypes reported within *All of Us*. Separated by genotype and electronic health records, our study seeks to (1) assess the genetic intersection of predicted rare pathogenic cancer predisposition variants identified by TCGA within the *All of Us* genomics cohort; (2) broaden our understanding of these predicted cancer predisposition variants on more diverse

[1]Department of Biology, Brigham Young University, Provo, UT 84061, USA; [2]Department of Chemical Engineering, Brigham Young University, Provo, UT 84602, USA; [3]Department of Microbiology and Molecular Biology, Brigham Young University, Provo, UT 84602, USA; [4]Department of Biomedical Informatics, Vanderbilt University, Nashville, TN 37203, USA; [5]Simmons Center for Cancer Research, Brigham Young University, Provo, UT 84602, USA
[6]Lead contact
*Correspondence: matthew.bailey@byu.edu
https://doi.org/10.1016/j.xhgg.2025.100405.
© 2025 Published by Elsevier Inc. on behalf of American Society of Human Genetics.
This is an open access article under the CC BY-NC-ND license (http://creativecommons.org/licenses/by-nc-nd/4.0/).

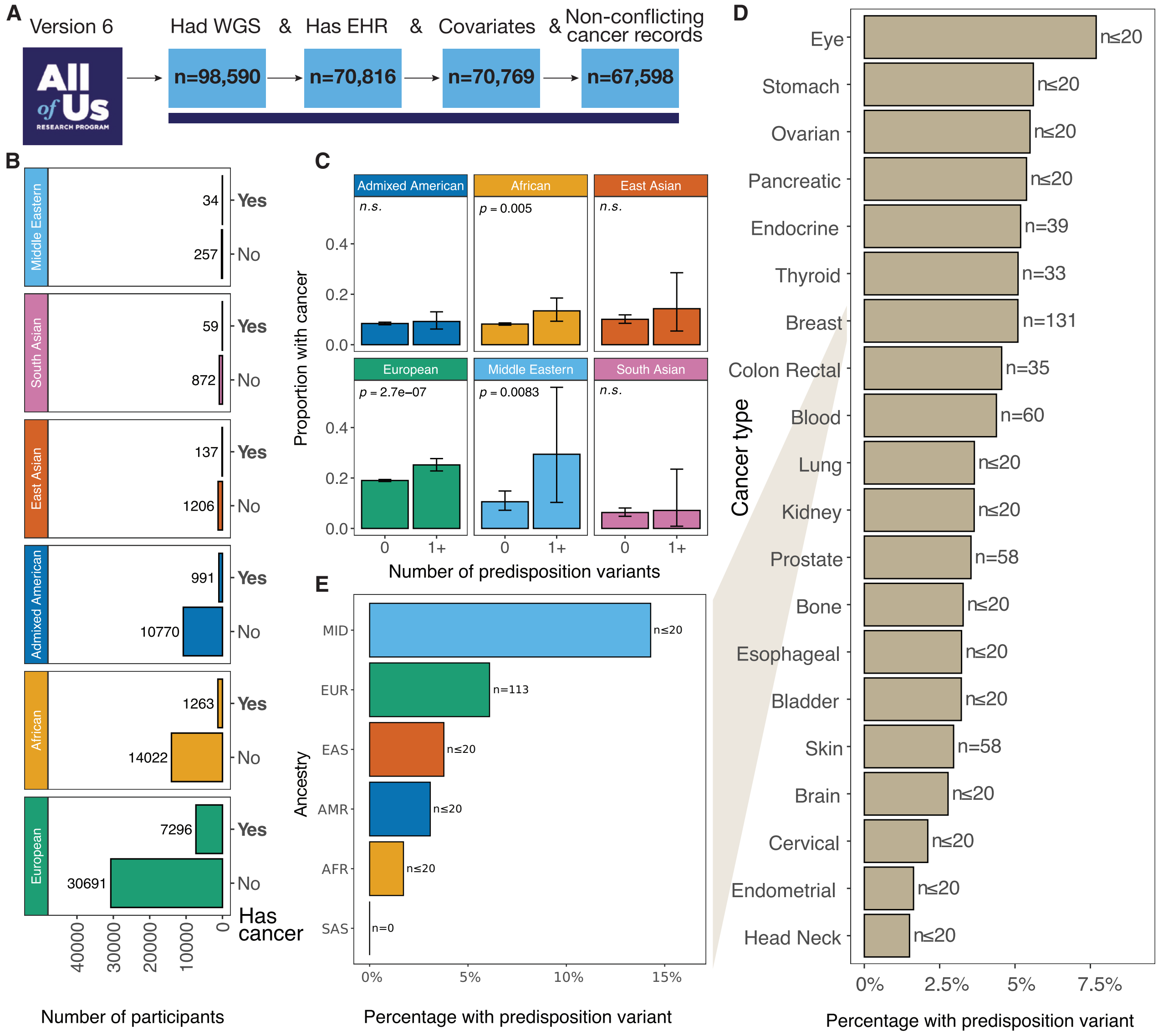

Figure 1. Prevalence of cancer incidence in the All of Us Research Program

(A) Manuscript overview that highlights data filtering and inclusion criteria.

(B) Bar plots indicate the number of samples in six broad groups of genetic similarity in *All of Us* that have a cancer phenotype ("Yes" or "No").

(C) Bar plots indicate the fraction of samples with cancer in the *All of Us* data with and without a cancer predisposition variant. These fractions are shown for each genetic similarity group. *P* values were calculated using logistic regression (corrected for age, sex, and the first 16 principal components). Error bars indicate 95% confidence intervals for the true proportions.

(D) Cancer codes were subdivided into 20 major organ systems. The x-axis indicates the proportion of those samples with previously reported pathogenic variants. The number of samples with a pathogenic variant is reported next to each bar.

(E) Breast cancer codes were analyzed by genetic ancestry. The x-axis indicates the proportion of those samples with previously reported rare pathogenic variants. The numerator of that proportion is reported next to each bar. Following *All of Us* protocols, all sample sizes less than or equal to 20 samples are reported as $n \leq 20$.

genetic ancestries, i.e., non-European; and (3) outline the overall power to identify new biomarkers of cancer as the *All of Us* Research Program grows.

Material and methods

Study population

This study utilized the data available in the *All of Us* Research Program Curated Data Repository (CDR, Controlled Tier Dataset v6). This dataset contains 372,397 participants, of whom 98,590 have short-read whole-genome sequencing (WGS) data (see Figure 1A). Of these individuals, 70,816 have insurance billing code data using SNOMED CT and International Classification of Diseases (ICD) criteria. We further restricted our study population to individuals with available information for sex at birth and age, leaving 70,769 individuals. Finally, we removed 3,171 people with conflicting cancer information (see the phenotyping section) to create a cohort of 67,598 individuals for our analyses. Age for each participant was defined as the participant's age at the cutoff date

Box 1. SNOMED cancer-code selection

Modifications to Aschebrook-Kilfoy cancer definition (SNOMED codes)
Updated code for endometrial cancer (93781006 instead of 10708511000119100)
Included skin cancer (94047004)
Removed specific head/neck cancer (372123001) since it is included in parent skin cancer code
Excluded individuals with no cancer codes but that reported having cancer in the *All of Us* Personal and Family Health History Survey

(January 1, 2022) for data in the *All of Us* version 6 (v6) data release.

Genomic data quality control and variant calling

The *All of Us* v6 Workbench provides access to these controlled data pending the completion of the *All of Us* data protection and orientation modules. Variant calls from *All of Us* were called using GATK germline best practices.[11,12] The *All of Us* bioinformatics team has reported a full list of quality control (QC) measures, which were performed on all individuals with WGS data according to sequencing protocols.[12] Briefly, highlights from this report include sample QC with call rates >98%, cross-individual contamination rates <0.03, and coverage requirements ≥30x mean coverage, ≥90% of bases with at least 20X coverage, ≥80 billion Q30 bases, and greater than or equal to 95% at 20x coverage in the following genes reported by the American College of Medical Genetics and Genomics (ACMG59) to be reported as incidental findings: *ACTA2, ACTC1, APC, APOB, ATP7B, BMPR1A, BRCA1, BRCA2, CACNA1S, COL3A1, DSC2, DSG2, DSP, FBN1, GLA, KCNH2, KCNQ1, LDLR, LMNA, MEN1, MLH1, MSH2, MSH6, MUTYH, MYBPC3, MYH11, MYH7, MYL2, MYL3, NF2, OTC, PCSK9, PKP2, PMS2, PRKAG2, PTEN, RB1, RET, RYR1, RYR2, SCN5A, SDHAF2, SDHB, SDHC, SDHD, SMAD3, SMAD4, STK11, TGFBR1, TGFBR2, TMEM43, TNNI3, TNNT2, TP53, TPM1, TSC1, TSC2, VHL,* and *WT1*. The WGS data in *All of Us* is based on the human genome reference build GRCh38.[13]

The *All of Us* research consortia provide QC reports with each data release. While these are available broadly to the public at https://allofus.nih.gov/, basic genomic QC metrics are provided here. All genomic centers used the same protocol for library construction (PCR Free Kapa HyperPrep), sequencer (NovaSeq 6000), software (DRAGEN v3.4.12), and software configuration. Any failure of the following metrics results in the exclusion of the sample. A sample is excluded if a sample swap is detected, identification of cross-contamination, or sample preparation errors. Next, all samples must meet the following criteria, (1) "Sex call is concordant with self-reported sex at birth or self-reported sex at birth reported as 'Other' or was not reported," (2) array call rates are greater than 98%, (3) WGS cross-contamination rates <3%, Cover, (4) mean coverage ≥30x, (5) ≥90% of bases covered at 20%, and (6) ≥95% at 20x in regions of the 59 *All of Us* Hereditary Disease Risk genes mentioned above.

Genetic ancestry and principal components

We acknowledge that there are inherent limitations and potential concerns when incorporating population descriptors, such as genetic ancestry groups, in research. This statement is especially true for broad, continental-level descriptors. Our aim in this study was to highlight the important work of *All of Us* to properly build a dataset for studying health challenges that reflects the rich genetic diversity in our society and the challenges we may face when relying on previous findings. For this study, we used the genetic ancestry and principal component data (including ancestry clusters) provided by *All of Us*. Briefly, the principal components were generated using all 98,590 individuals with WGS data. Based on principal component clustering, individuals were given a likelihood of belonging to each of six predominant genetic ancestry groups: African/African American (AFR), Admixed American/Latino (AMR), East Asian (EAS), European (EUR), Middle Eastern (MID), or South Asian (SAS). These categories were taken from those used by gnomAD, the Human Genome Diversity Project, and 1000 Genomes. Individuals were then assigned to a single ancestry corresponding to the highest likelihood. *All of Us* also provides an "Other" label for individuals not belonging clearly to one ancestry, but we did not incorporate this information in our analyses in order to maximize the sample sizes of the six predominant ancestry groups. These genetic ancestries reflect genetic similarity among individuals at a broad continental level and should not be interpreted to mean race, ethnicity, or individual identity. Additionally, to emphasize that these six groups are simplifications of any individual's true genetic ancestry, we discuss individuals as being "AFR-like," "AMR-like," "EAS-like," "EUR-like," "MID-like," or "SAS-like" throughout the paper.

Sex at birth

To determine each individual's sex at birth, we first used the "impute_sex" function provided by Hail with the default minor allele frequency (MAF) cutoff of 0.05 on the sex chromosomes (https://github.com/hail-is/hail/releases/tag/0.2.13). This function calculates an F-score that ranges from 0 (female) to 1 (male) and assigns a sex based on this score. We used Hail's default thresholds of $F < 0.2$ for females and $F > 0.8$ for males. F-scores between 0.2 and 0.8 are considered inconclusive and assigned a missing value for sex ($n = 6124$). We then compared the imputed sex with the self-reported sex from *All of Us* to determine each person's sex at birth. Any individuals whose imputed sex differed from the self-reported sex at birth were excluded ($n \leq 20$ of the 98,590 with WGS data). If one of the two values (imputed or self-reported) was missing for a particular individual, we accepted the non-missing value as their sex at birth.

Phenotyping

Cancer cases and controls were identified from EHR data using the SNOMED codes provided by Aschebrook-Kilfoy et al., with slight modifications (see below, Box 1).[14] Any individual with at least one SNOMED cancer code was considered a cancer case. We excluded 3,171 individuals who reported having cancer in the

All of Us survey but had no SNOMED cancer codes, leaving 67,598 individuals in our study population.

Additional phenotypes were determined by converting International Classification of Diseases (ICD)-9 and ICD-10 codes to phecodes using the PheWAS package (v0.99.6–1)[15] with Phecode Map 1.2 available for R version 4.3.1 "Beagle Scouts." (This version was used throughout this project.) This package maps all ICD-9 and ICD-10 codes to one of 1,866 defined phecodes. Individuals with at least two instances of a phecode occurring on unique days are considered cases for that phecode. Each phecode is placed into one of 17 groups (e.g., infectious diseases, symptoms, etc.) as defined by the PheWAS package.

Predisposition variants

All of Us v6 provides the WGS data as a Hail MatrixTable. We first filtered out any variants from the MatrixTable that were flagged as "PASS" or "." from the Hail Table. Specifically, hard threshold filters were applied and displayed in the FILTER field of the Hail MatrixTable. From *All of Us* documentation, "Unfiltered variants will have '.' or 'PASS' in the FILTER field in the WGS joint callset VCFs and Hail MT. We recommend that researchers do not include sites that were filtered in their analyses." Additional descriptions of these "hard threshold filters" are described below and in the All of Us Genomics Quality Report, "If a variant does not meet the following criteria, it will be filtered (i.e., a value will appear in the FILTER field of VCFs and Hail MatrixTables (MT)). These were based on the following criteria:

(1) No high-quality genotype (GQ>=20, DP>=10, and AB>=0.2 for heterozygotes) called for the variant. Note that, 'Allele Balance (AB)' is calculated for each heterozygous variant as the number of bases supporting the least-represented allele over the total number of base observations. In other words, min(AD)/DP for diploid GTs. This resulted in the following FILTER field as 'NO_HQ_GENOTYPES.'
(2) 'ExcessHet' < 54.69. Not the ExcessHet is a phred-scaled *p* value. The *All of Us* genomic coordinators use the cutoff of any value more extreme than a *Z* score of −4.5 (*p* value of 3.4e−06). This equates to a phred-scaled value of 54.69.
(3) 'QUAL' score is too low (lower than 60 for SNPs; and 69 for Indels). THE QUAL tells you how confident we are that there is some kind of variation at a given site. The variation may be present in one or more samples. The FILTER field value is 'LowQual'"

We then restricted the dataset to include only the 586 cancer predisposition variants (across 99 genes) from TCGA, as reported by Huang et al. The Lift Genome Annotations tool[16] provided by UCSC was used to convert these TCGA variants from GRCh37 to GRCh38.

Cancer occurrence analysis

Individuals were labeled as cancer cases or controls, as described above. Individuals with at least one variant allele from any of the cancer variants (586 variants across 99 genes) reported by Huang et al. were labeled as having a predisposition variant, with the remaining individuals labeled as genomic controls.[7] Logistic regression was then performed for each ancestry to compare cancer occurrence against the presence of a predisposition variant with sex at birth, age, and the 16 principal components provided by *All of Us* as covariates. The significance level was set at $\alpha = 0.05$ for each test.

Age at onset analysis

For individuals labeled as cancer cases, we estimated the diagnosis date as the earliest cancer EHR occurrence. We then calculated age at diagnosis using birth date, considering age at diagnosis to be a rough estimate for the age of cancer onset. As in the cancer occurrence analysis, each individual with at least one predisposition allele was labeled as having a predisposition variant, with the remaining individuals labeled as controls. For each ancestry, we then compared age at diagnosis between individuals with and without predisposition variants using a Cox proportional hazards analysis in the R Survival package.[17] In addition to predisposition variant status, we included sex at birth and the 16 principal components provided by *All of Us* as covariates. The significance level was set at $\alpha = 0.05$ for each of the six tests (one for each ancestry).

Predisposition gene PheWAS

Two related analyses are described here: a traditional phenome-wide association study (PheWAS) and a cancer-specific association study. Each analysis was run twice—once with only EUR-like individuals and a second time with individuals from the other five genetic ancestries (AFR, AMR, EAS, MID, and SAS). We combined the five non-EUR ancestries (AFR, AMR, EAS, MID, and SAS) due to their smaller sample sizes, especially regarding the number of individuals with cancer and the number with predisposition variants. In particular, combining these ancestries was necessary to have enough people with predisposition variants for each association study. Due to the low frequency of the predisposition variants in *All of Us*, we grouped all variants by gene for these analyses. Out of 99 genes with variants, we only included the seven that had at least 20 individuals with a predisposition variant in each of the two broad ancestry groups (EUR and non-EUR): *BRCA2* (MIM: 600185), *BRIP1* (MIM: 605882), *FAH* (MIM: 613871), *SH2B3* (MIM: 605093), *GJB2* (MIM: 121011), *RECQL4* (MIM: 603780), and *WRN* (MIM: 604611). We first performed logistic regression for each broad ancestry group to compare the presence of variants in these genes to each of the cancer types we identified using SNOMED codes. We then ran a traditional PheWAS analysis for each broad ancestry group with methods provided in the R PheWAS package.[15] As a note, 1,220 people were left out of the traditional PheWAS (leaving 66,378 individuals) due to only having EHR records based on classification systems other than ICD-9 and ICD-10. We included age, sex at birth, and all 16 principal components for genetic ancestry provided by *All of Us* as covariates in both analyses. There were 294 tests in the cancer-specific analysis (2 ancestry groups × 7 genes × 21 cancer types), so we set the significance threshold at $\alpha = 1.7 \times 10^{-4}$. The traditional PheWAS included 25,942 tests (2 ancestry groups × 7 genes × 1853 phecodes), so we set $\alpha = 1.9 \times 10^{-6}$ for that analysis.

Variant distribution analysis

To assess the distribution of variants across the six genetic ancestries, we calculated the number of times variant alleles (of the 586 variants reported by TCGA) were found in each ancestry. We then tested the null hypothesis that variant alleles were equally distributed across ancestries with a chi-square goodness-of-fit test. Under this hypothesis, the observed proportion of alleles in a specific ancestry should be close to the proportion of total people from the ancestry in our study population (e.g., if 56% of our dataset is EUR, then ∼56% of variant alleles should come from EUR participants). While AFR individuals have been shown to carry more genetic variation in general,[7] we did not adjust the null hypothesis accordingly given that we were assessing a specific set of rare

Table 1. Summary information for the study population of *67,598* individuals

Genetic ancestry	Sample size	Cancer cases	People with predisposition variant	Mean age (age range)	Female/Male
European	37,987	7,296	1,243	58.6 (18.5–103.5)	23,091/14,896
African	15,285	1,263	231	51.8 (19.5–99.5)	9,709/5,576
Admixed American	11,761	991	304	48.0 (18.5–101.5)	8,309/3,452
East Asian	1,343	137	42	48.2 (19.5–91.5)	910/433
South Asian	931	59	45 (grouped to mask count <20)	46.6 (19.5–86.5)	514/417
Middle Eastern	291	34		51.1 (20.5–93.5)	157/134
Total	67,598	9,780	1,865	54.8 (18.5–103.5)	42,690/24,908

pathogenic variants and not common variants driving genetic variation. Thus, interpretation of the p value from this test would not suffice if rare pathogenic mutations were more common in AFR individuals, but this was not the case for any rare pathogenic mutations. We then performed a binomial test for each individual ancestry to see which ancestries had more/less variant alleles than expected. Finally, we examined the distribution of variants across ancestries on a gene-by-gene basis. After counting the number of variant alleles in each ancestry for each predisposition gene, we performed a series of binomial tests to compare the observed proportions of variant alleles against the expected proportions (based on the total number of people from each ancestry as before). There were a total of 474 tests (79 genes × 6 ancestries) in this gene-by-gene analysis, so we set $\alpha = 1.05 \times 10^{-4}$.

Power calculations

GWAS power calculations were derived from the work of Skol et al.[18] To briefly summarize, the power of a GWAS analysis depends on several variables: sample size, the frequency of the phenotype being analyzed, the effect size of any particular variant, allele frequency, and the significance threshold. We set our significance threshold to the standard value for genome-wide significance ($p = 5 \times 10^{-8}$). We calculated the power of a GWAS analysis in *All of Us* across a range of values for both relative risk (RR) and minor allele frequency (MAF). Though this study focuses on rare variants (MAF $\leq$0.01), we included higher allele frequencies (MAF >0.01) to make these estimates applicable to studies with aggregated variants and common variants. To aid in choosing appropriate RR and MAF values for our power estimates, we determined the RR of cancer occurrence for individuals carrying a pathogenic variant compared with non-carriers across all ancestries (RR = 1.44). We also calculated RR for each ancestry, and the resulting values ranged from 1.10 to 2.78. Assuming that common variants are generally less pathogenic than rare variants, we based power calculations for common and less-common variants (MAF >0.01) around the lowest RR estimate of 1.10. For rare variants (MAF $\leq$0.01) we based our power calculations on the overall estimated RR of 1.44.

Results

Data overview

In the *All of Us* v6 data release, we found 67,598 individuals who met the basic data requirements for this study (whole-genome sequencing, EHRs, and age, Figure 1A; Table 1) (see material and methods). For these individuals, there are a total of 20,394,417 billing codes available (median = 103; range = (1; 16,107)). In our cohort, we identified 9,780 (14.5%) individuals with malignant neoplasms (see material and methods, Figure S1; Table 1). Following the six broad groups of genetic similarity provided by *All of Us* (see material and methods), we observed most participants with malignant neoplasms were of European genetic ancestry (EUR, 74.6%). After EUR, the next largest groups are those of African/African American (AFR, 12.9%) and Admixed American/Latino (AMR, 10.1%) genetic ancestry, with relatively few coming from the remaining three: East Asian (EAS: 1.4%), South Asian (SAS: 0.6%), and Middle Eastern (MID: 0.3%, Figure 1B). The age distribution of participants varied widely by genetic ancestry (Figure S2A), and mean age showed a strong correlation with cancer occurrence (Figure S2B). Consequently, we adjusted for age in all subsequent analyses.

Pathogenic predisposition variants pancancer

EUR-like participants were among the most frequent groups with rare pathogenic predisposition variants, as reported by TCGA.[7] A total of 586 pathogenic variants (across 99 genes) were reported by TCGA. Still, only 280 variants (across 79 genes) were observed in our study population, likely because all of the reported variants were rare (MAF <1%). We found 1,865 individuals (2.8%) in our study population that carried one or more pathogenic variants. Once again, these individuals were primarily EUR-like (66.6%), with a small portion coming from other ancestries (AFR: 12.4%; AMR: 16.3%; EAS+SAS+MID: 4.7%). Note that due to the rarity of the predisposition variants, many counts will be displayed as "$\leq$20" to comply with the *All of Us* data dissemination policy. Additionally, some counts and percentages will be combined (as was done above) to mask counts less than 20 that could otherwise be calculated.

After identifying individuals with cancer and those with pathogenic predisposition variants, we calculated the overall effect of these variants on cancer occurrence and how that effect varies across ancestries. First, when taken collectively, we observed an upward trend in the proportion of cancer occurrence in individuals with predisposition variants across all ancestries (Figure 1C). This trend was

Table 2. Impact of pathogenic germline variants on cancer occurrence and timing

	Summary counts		Cancer occurrence		Age at onset			
			Predisposition variant		Predisposition variant		Sex at birth	
Genetic ancestry	Sample size	Cancer cases	p	Odds Ratio (95% CI)	p	Hazard Ratio (95% CI)	p	Hazard Ratio (95% CI)
European	37,987	7,296	2.7×10^{-7}	1.44 (1.26–1.66)	0.14	1.09 (0.97–1.22)	4.7×10^{-60}	0.68 (0.65–0.71)
African	15,285	1,263	0.0050	1.78 (1.19–2.66)	0.27	1.23 (0.85–1.77)	1.3×10^{-7}	0.73 (0.65–0.82)
Admixed American	11,761	991	0.38	1.20 (0.80–1.81)	0.45	0.86 (0.58–1.27)	5.1×10^{-6}	0.73 (0.63–0.83)
East Asian	1,343	137	0.39	1.51 (0.59–3.86)	0.82	1.11 (0.47–2.62)	0.0015	0.47 (0.30–0.75)
South Asian	931	59	0.72	1.33 (0.28–6.41)	0.73	1.77 (0.07–45.9)	4.1×10^{-4}	0.26 (0.13–0.55)
Middle Eastern	291	34	0.0083	6.80 (1.64–28.3)	0.96	0.96 (0.26–3.54)	0.98	1.01 (0.29–3.53)

statistically significant in three ancestries: EUR, AFR, and MID (Table 2). All other genetic ancestries had p values greater than 0.05.

Additionally, we investigated the potential effect of predisposition variants on the timing of cancer onset and whether this effect varied by genetic ancestry (Figure S2C). Overall, in the *All of Us* dataset, EUR-like individuals start having cancer-related EHR records at a later age compared with other ancestries. As mentioned previously, although the underlying age distributions are different for each ancestry, this result may simply reflect sample bias (Figure S2A). Predisposition variants failed to show a significant effect on age at cancer onset in any genetic ancestry (Table 2). Meanwhile, sex at birth had a significant effect on timing in each ancestry except MID (Table 2). The lack of a significant result for MID was likely due to a smaller sample size (only 34 individuals in the analysis). In all genetic ancestries except MID, females were younger, on average, than males at cancer onset. This finding is supported by previous research.[19]

Pathogenic variants by cancer's site of origin

The primary location of a tumor significantly contributes to cancer's biological and environmental heterogeneity.[20] We leveraged SNOMED codes to classify the site of origin for the participants with neoplasms as previously reported (see material and methods, Figure 1D).[14] We observed that ocular neoplasms had the highest proportion of samples with a cancer predisposition variant (>7.5%, ≤20 samples). On the other hand, head and neck tumors possess one of the lowest proportions of samples that carry predisposition variations (<2%, ≤20 samples). We estimated that ~5% of breast tumors carry a rare cancer predisposition variant, which is lower than a previous estimate of 10.2% in White women.[21] Upon deeper investigation, we observed large differences in the proportions of breast neoplasms carrying pathogenic variants between genetic ancestries (Figure 1E). In *All of Us*, SAS and AFR had the lowest fraction of breast cancer cases with predisposition variants, whereas EUR and MID had the highest fraction. The extreme proportions observed in MID and SAS are likely due to small sample sizes.

PheWAS with cancer predisposition genes

Following our investigation of the overall effects of the TCGA pathogenic variants on cancer occurrence and timing, we took a closer look at the relationship between individual genes and specific phenotypes. Since most of the variants we found in the *All of Us* dataset were seen in only a handful of individuals, our ability to assess the effects at the variant level was limited (Table S1; Figure S3). Consequently, we included pathogenic variants (grouped by gene, i.e., pathogenic genes) from only seven genes in our subsequent analyses (material and methods, Figure 2). As a first pass, we looked at the cancer types identified using SNOMED codes (material and methods, Figure 2A; Table S2). Six associations passed the significance threshold of $p = 1.7 \times 10^{-4}$ (Bonferroni correction for 294 tests), five of which were linked to *BRCA2*. The most prominent of these was the connection between *BRCA2* and breast cancer in EUR-like participants ($p = 1.0 \times 10^{-15}$, odds ratio [OR] = 11). This association was also significant in non-EUR-like participants ($p = 4.1 \times 10^{-6}$, OR = 14). Another prominent result that showed up in both the EUR and non-EUR groups was between *BRCA2* and ovarian cancer (EUR: $p = 1.6 \times 10^{-8}$, OR = 13; non-EUR: $p = 1.0 \times 10^{-5}$, OR = 33). The links between breast/ovarian cancer and *BRCA2* are well supported by literature.[22,23] The only significant association unrelated to *BRCA2* was between colorectal cancer and *SH2B3* in EUR-like individuals ($p = 1.2 \times 10^{-4}$, OR = 5.3).

After this cancer-specific analysis, we broadened our scope to include a wide range of phenotypes using ICD-9 and ICD-10 codes. The PheWAS with EUR-like individuals had a total of 13 associations that passed the significance threshold of $p = 1.9 \times 10^{-6}$ (Bonferroni correction for 25,942 tests) (Figure 2B; Table S3). The majority of these hits were neoplasm-related and were associations between *BRCA2* and breast/ovarian cancer, confirming the previous results (Figure 2A). Additionally, this finding agrees with the original data reported in TCGA whereby the majority (55%) of pathogenic *BRCA2* variants occurred in patients with breast or ovarian cancer. Another gene also had a significant association with a neoplasm-related phecode: *BRIP1* with phecode 209 ("Neuroendocrine tumors,"

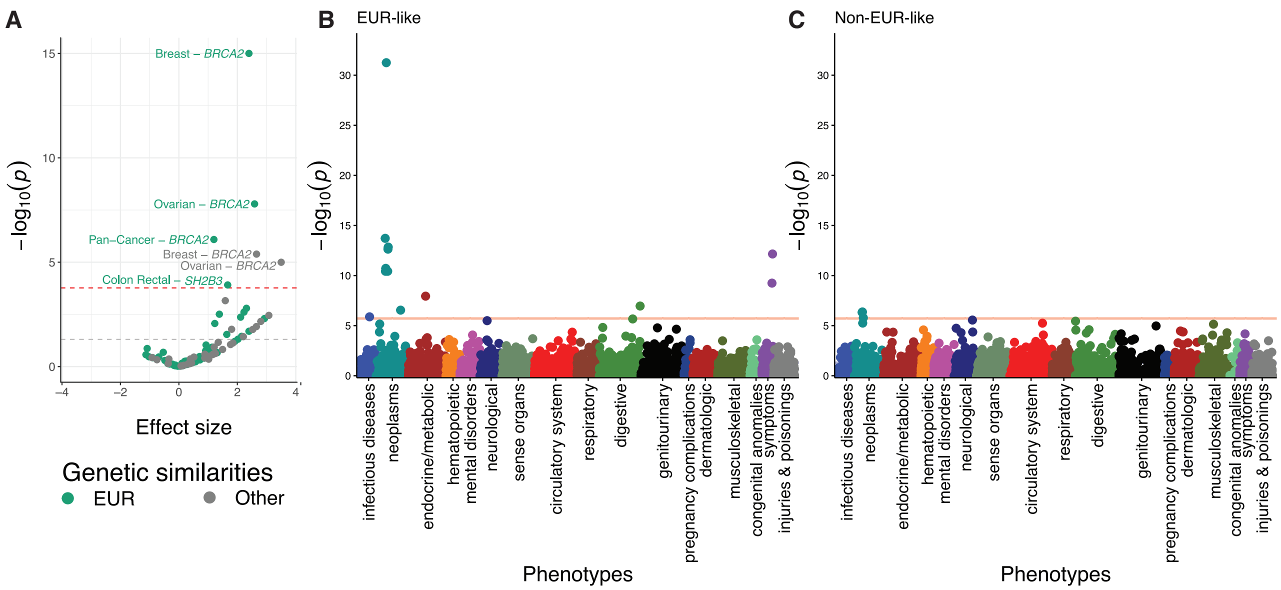

Figure 2. PheWAS using *All of Us* and seven cancer genes
(A) Volcano plot establishes genomic relationships between variants in seven cancer genes and SNOMED cancer codes. Logistic regression was corrected for age, sex, and the first 16 principal components (−log10 *p* value on the y axis, and effect size estimates on the x axis). The red horizontal line indicates the alpha threshold corrected for the number of analyzed cancer types and genes ($\alpha = 1.7 \times 10^{-4}$).
(B and C) A Manhattan Plot separated by phenotypic categories (x axis) establishes genomic relationships between variants in seven cancer genes and ICD-9 and ICD-10 insurance billing codes (−log10 *p* value on the y axis). (B) Displays PheWAS results for a cohort containing only participants of European-like genetic ancestry (EUR-like). (C) Displays PheWAS results for a cohort containing all other genetic ancestries (non-EUR-like). The red horizontal line indicates the alpha threshold for the number of phenotypes and genes analyzed ($\alpha = 1.9 \times 10^{-6}$).

$p = 2.8 \times 10^{-7}$, OR = 24). Even outside of the neoplasms group, several of the significant hits are for phenotypes that can potentially be linked to cancer—“abnormal tumor markers” (phecode 795.8), “postablative ovarian failure” (phecode 258.1), and “hemorrhage of gastrointestinal tract” (phecode 578.9), for example.

The PheWAS in other ancestries had a different set of results (Figure 2C). Notably, there were only three statistically significant results, though all three were associations between *BRCA2* variants and breast cancer. Additionally, the increased presence of *BRCA2* variants in ovarian cancer from the cancer-specific analysis showed trending significance in the traditional PheWAS (phecode 184.11, “malignant neoplasm of ovary”, $p = 1.6 \times 10^{-3}$, OR = 32) (Figures 2A and 2C). Though many of the significant associations from the EUR PheWAS failed to reach significance in the non-EUR PheWAS, the effect sizes for these gene-phenotype associations were similar between the two groups (Figure S3). This suggests that the contrast between the two groups seen in Figures 2B and 2C may reflect factors other than variant pathogenicity. Such factors may include differences in sample size, variant frequency, or possible modifier alleles between ancestries (Figure 3).

Observations of specific cancer predisposition genes

SH2B3, SH2B adaptor protein 3, codes for a protein that is involved in many growth factors and specifically is a negative regulator of cytokine signaling. The most frequently mutated amino acid ($n = 180$, Table S1) is *p.(Glu208Gln)* and was labeled in 2010 as a pathogenic somatic variant by ClinVar (https://www.ncbi.nlm.nih.gov/clinvar/RCV000023398/). However, ClinVar recently reported this mutation in May 2023 as likely benign. Here we see similar mutation frequencies between AMR-like and EUR-like participants (Figure 3). An in-depth assessment of EUR-like individuals suggests that this variant is frequently observed in colon and rectal tumors (p value = 1.23×10^{-4}, OR = 5.31), which supports the likely pathogenic classification from 2010 despite being a germline and not somatic variant (as reported, Figure 2A). On the contrary, when non-EUR-like individuals were analyzed, no significant difference was observed in participants that carry the *p.(Glu208Gln)* variant and colorectal cancer occurrence (p value = 0.96, OR = 8.15×10^{-6}). Thus, this variant in non-EUR-like individuals has negligible impact on the phenotype, consistent with the more recent classification of likely benign. Importantly, the number of people carrying this variant in *SH2B3* is similar between the EUR ($n = 92$) and non-EUR ($n = 88$) groups, indicating similar power in both assessments.

Another gene with a high frequency of predicted pathogenic variants was *GJB2*, gap junction protein beta 2, which encodes a connexin protein. Overexpression of *GJB2* is associated with a poor prognosis in several human cancers.[24,25] The *p.(Glu47Ter)* mutation has been reported by ClinVar to be pathogenic since 1998 and is typically associated with autosomal recessive nonsyndromic hearing loss 1A (DFNB1A).[26] Interestingly, we observed

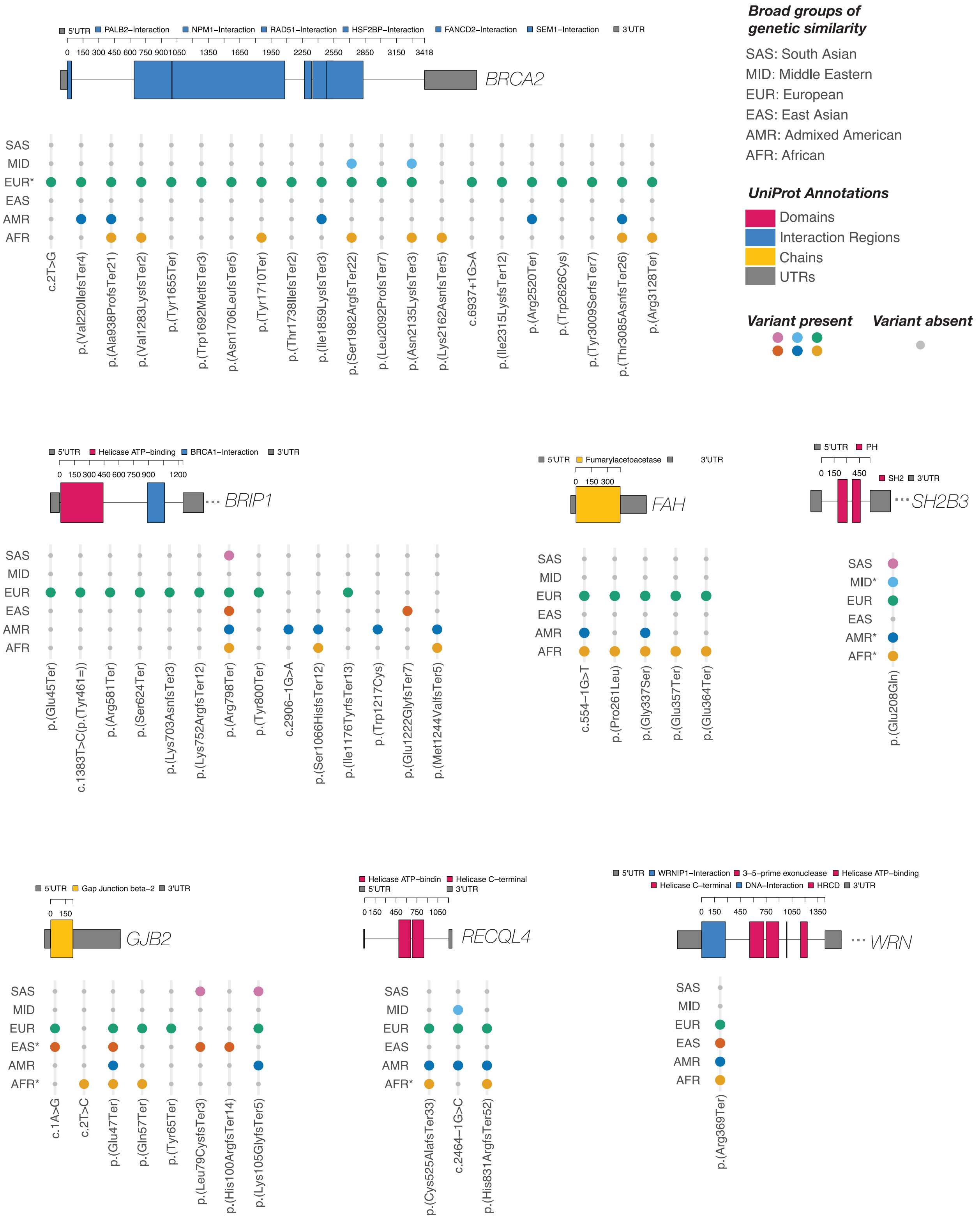

Figure 3. Fraction of cancer predisposition mutations

Dot plots display the variants in the seven genes used for the PheWAS analysis. If a dot is colored, then that variant was identified in at least one participant of that ancestry. Gene models are displayed above the genes. Domains are colored in red and protein interaction regions are colored in blue. When Uniprot did not provide interaction regions or domains then "Uniprot-chains" are displayed in yellow. "…" indicates truncated UTRs on the gene model. The asterisk next to ancestry labels signifies a significant difference from expected proportions.

trending significance in EUR-like participants for pancreatic cancer (p value = 4.29×10^{-3}, OR = 8.26) and ocular tumors (p value = 4.99×10^{-3}, OR = 18.7) (Figure 2A). On the other hand, non-EUR-like participants showed some association with increased prevalence of colorectal tumors (p value = 0.016, OR = 6.07). While these results provided limited support for the current pathogenic classification, we anticipate that as the *All of Us* Research Program reaches 1 million participants, we will be powered to clarify the association of these rare pathogenic mutations in *GJB2* to cancer predisposition.

Distribution of rare pathogenic variants across ancestries

The contrasting PheWAS results between the EUR and non-EUR groups led us to examine the distribution of variants across genetic ancestries (Figure 3). Of the 53 variants from TCGA across the seven genes included in the PheWAS analysis, EUR-like participants had the highest fraction of variant alleles in 36 (67.9%) variants. The next highest count (AFR: 9 variants, 17.0%) was much lower. For 19 (35.8%) variants, EUR was the only ancestry where a variant allele was found. Meanwhile, there were nine (17.0%) variants that were only found in non-EUR-like individuals. Furthermore, we saw that broadly, the variants from all predisposition genes did not distribute evenly across genetic ancestries (p value = 4.2×10^{-29}, chi-square test). Binomial tests for each ancestry revealed more variant alleles than expected in EUR-like participants (p value = 2.0×10^{-22}) and fewer than expected in AFR-like participants (p value = 8.1×10^{-31}). These numbers show that the variants from TCGA are enriched in EUR-like individuals,[8] which may have contributed to the lack of significant PheWAS results for non-EUR-like participants. Two other important factors, though, should also be considered: (1) most of these variants occur in only a handful of individuals, which lends itself to more extreme proportions and (2) the TCGA dataset, where the variants were identified, is composed primarily of individuals who self-reported their race as White.[27] Regardless, the scarcity of pathogenic variants in non-EUR-like participants limited our ability to validate their effect in diverse ancestries.

Though the overall numbers point toward a disproportionately large number of rare variant alleles coming from EUR-like individuals (Figure 3), individual genes vary in their distributions. The genes with fewer variants, such as *SH2B3*, *FAH*, *RECQL4*, and *WRN*, exhibit a more balanced spread of variant alleles across ancestries (Figure 3). Importantly, none of the variants in these genes are found in only one ancestry. Using binomial tests, we identified ancestries with significantly more or less variant alleles than expected in each of the predisposition genes from TCGA (Figure 3). Of note, EAS-like participants carried more variants than expected by chance in *GJB2* (p value = 7.10×10^{-15}), while AFR-like participants exhibited fewer variants than expected in *SH2B3* (p value = 3.30×10^{-9}), *GJB2* (p value = 4.64×10^{-6}), and *RECQL4* (p value = 5.48×10^{-5}). Additionally, EUR-like participants had significantly more predisposition variants in BRCA2 than expected by chance (p value = 1.26×10^{-5}). In fact, almost half of the *BRCA2* variants (10 of 21) were found only in EUR-like individuals. This fact is noteworthy since most of the significant cancer-related results from the PheWAS analyses were tied to *BRCA2* (Figure 2). Taken together, these results confirm intrinsic difficulties in comparing the effect of predisposition variants across genetic ancestries due to an unequal distribution of variants.

Power calculations: Moving forward

While the *All of Us* Research Program has gained traction within the health and disease research community, it has yet to reach its goal of 1 million whole-genome samples.[11,28,29] To contextualize our findings, we performed broad power calculations to predict the power we will have in follow-up cancer analyses using these data. Using a GWAS framework, we estimated statistical power to identify cancer-associated variants in the projected 1 million samples at varying allele frequencies and relative risk (RR) scores. Starting with relatively common MAF values, we observed sufficient power to detect significant mutations when RR was near 1.2 (material and methods, Figure 4A). Setting RR value of 1.2 and minor allele frequency (MAF) of 0.05, we estimate >99% power to detect significant GWAS findings using the projected sample sizes of *All of Us* (material and methods, Figure 4A). In comparison, we estimate only 11.9% power to detect cancer-associated variants in our current study population (67,598 individuals). This current lack of power is exacerbated when assessing individual genetic ancestries (EUR: 1.2%; AFR 0.031% Figure 4A). An MAF of 0.05 far exceeds the MAFs reported here where the rarest pathogenic variants we can report on is 0.0001 (20/(2 × 67,598), using the *All of Us* 20 participant limit). The most frequently mutated variant we observed from TCGA pathogenic variants was the *p.(Glu208Gln)* mutation in *SH2B3*, which had MAF = 0.0013 of individuals (180/(2 × 67,598)).

We extended our power analysis to capture a wide range of allele frequencies (Figures 4B and 4C). Of particular interest to this study were rarer allele frequencies and higher effect sizes to match observed values (MAF <0.002 and RR = 1.44, Figure 4C). By extrapolating the current ratios of various ancestries to 1 million total participants, we estimate that our power to detect rare mutations (0.001 MAF) with high effect sizes (RR = 1.44) will be 28.8% with all participants, and much lower power if assessing individual genetic ancestries (3.7% in EUR-like participants, and 0.089% in AFR-like participants, Figure 4C). These numbers indicate limited power to identify rare cancer-associated variants with GWAS-style analysis, even as *All of Us* approaches its goal of 1 million participants. However, we eagerly await the growth of the *All of Us* dataset over time and its potential to increase our understanding of cancer-associated germline variants.

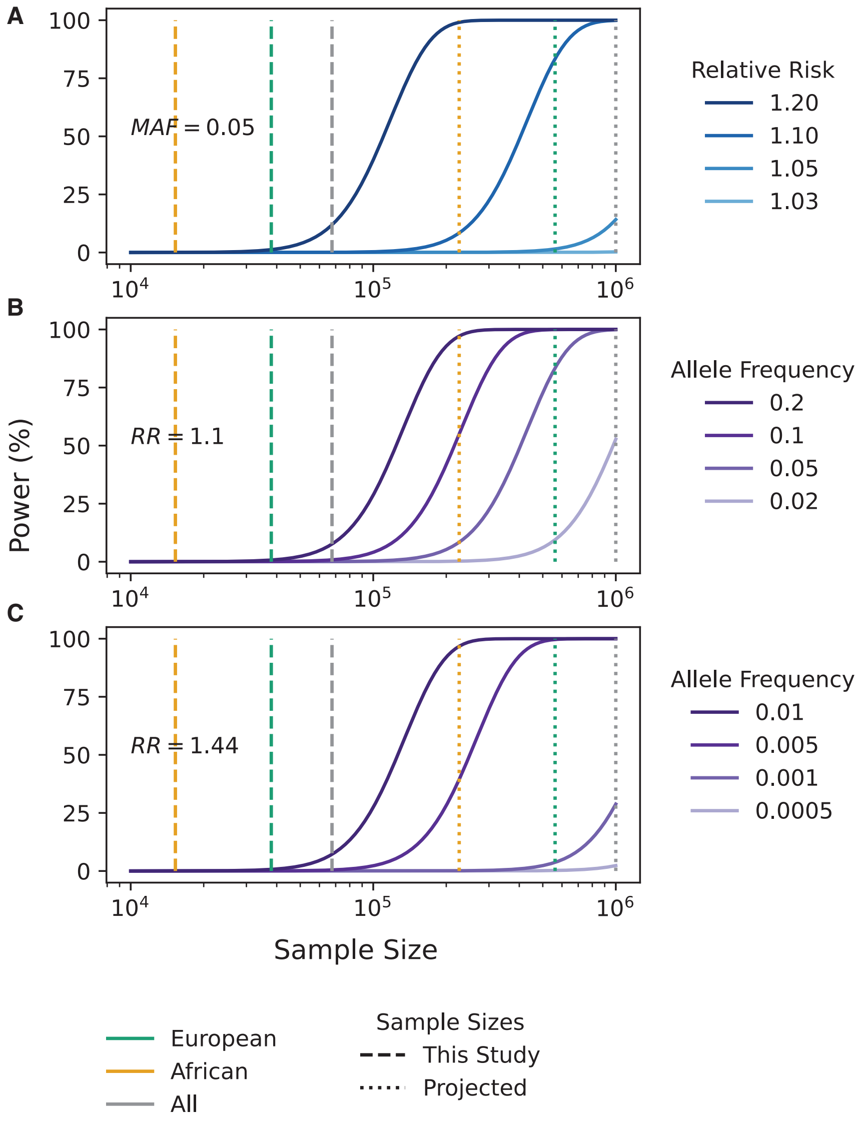

Figure 4. Power calculation estimates for *All of Us* GWAS studies

Power curves (percent power on the y axis and sample size on the x axis) are displayed.

(A) Using an MAF of 0.05 we show the power to detect variant alleles with various effect sizes in a GWAS analysis. Current and projected sample sizes in *All of Us* are shown with vertical dashed lines for all ancestries combined or for European/African genetic ancestries individually.

(B) Power to detect common variants (MAF $\geq$0.02) with a smaller effect size (RR = 1.1) is shown. Again, current and projected sample sizes are shown with vertical lines.

(C) Same as (B) but for less-common/rare variants (MAF $\leq$0.01) with a higher effect size (RR = 1.44).

Discussion

Billions of federal taxpayer dollars have given the world an unprecedented glimpse into the power of genomics to predict health outcomes.[30,31] The successful TCGA cohort reinforced our understanding of known genes and pathways impacting cancer phenotypes and proposed many new genes that alter cancer trajectories.[32] Here, using the *All of Us* v6 cohort, we validated some key germline-specific TCGA findings. First, we show that neoplastic phenotypes are prevalent in the dataset and that some cancer phenotypes significantly correlate with known rare predisposition variants from TCGA. Notably, however, many of the cancer predisposition variants failed to show a significant effect when analyzed in participants of non-European genetic ancestry. This study adds to the growing literature that justifies the creation of more diverse data repositories like the *All of Us* Research Program.[33–36] It also confirms the hesitation that is necessary when applying results from previous studies to more diverse data collections.[34,37]

This body of research is to be taken in the context of its limitations. First, *All of Us* continues to acquire more participants. While our study includes data from 67,598 participants, it does not currently reflect the entirety of the *All of Us* cohort.[9] The *All of Us* workbench will continue to expand to obtain genomic and EHR data for 1 million individuals. We fully anticipate that future versions of this work will provide more accurate estimates of the impact of rare pathogenic germline mutations on cancer predisposition in the United States. While we anticipate limited discrepancies between this study and the future version, we do anticipate continued improvements in statistical power. We performed a power analysis predicting our performance with estimates of 1 million participants.

Having a large genomic dataset presents strong invitations to try to identify new genetic associations to cancer. However, discovering new cancer predisposition variants was not the focus of this paper for many reasons. First, our power to detect novel cancer predisposition variants was limited by the number and age distributions of the participants in the *All of Us* cohort. Second, our primary objective was to leverage existing data from one of the largest cancer datasets (TCGA) and compare their findings to diverse genetic populations. As the *All of Us* Research Program grows to 1 million participants, we will have increased power to detect significant genome-wide associations with cancer if the mutations are common (MAF >0.02). As studied here, however, rare pathogenic variants will continue to require alternative strategies for identification and association with disease despite the large sample sizes. As Huang et al. and many others have performed,

predictive pathogenicity algorithms coupled with a cancer-rich dataset will be needed to associate predisposition mutations and cancer risk.[7]

In looking to the future of large integrative data models, we, the authors, are compelled to compliment the structure and intention behind the construction of the *All of Us* workbench. Because of the extensive efforts of the *All of Us* research teams, the United States now has a population database to investigate health records. We also applaud this centralized data model as a template for future national and international genomic datasets. The *All of Us* workbench is positioned to improve reproducibility and democratize existing silos of genomic expertise. As more and more users come to this resource, we hope our shareable pipelines will expedite efforts to find new associations and cures to human disease. While versioning continues to change, we have built a robust pipeline for the *All of Us* v6 Workbench to assess the consequences of germline mutations on cancer predisposition.

Data and code availability

All analyses were done using Jupyter Notebooks on the *All of Us* workbench. Following controlled access registration and training, we will make our notebooks available upon request through the *All of Us* workbench.

Acknowledgments

The content is solely the responsibility of the authors and does not necessarily represent the official views of the National Institutes of Health. The *All of Us* Research Program is supported by the National Institutes of Health, Office of the Director: Regional Medical Centers: 1 OT2 OD026549, 1 OT2 OD026554, 1 OT2 OD026557, 1 OT2 OD026556, 1 OT2 OD026550, 1 OT2 OD 026552, 1 OT2 OD026553, 1 OT2 OD026548, 1 OT2 OD026551, 1 OT2 OD026 555; IAA #: AOD 16037; Federally Qualified Health Centers: HHSN 263201600085U; Data and Research Center: 5 U2C OD023196; Biobank: 1 U24 OD023121; The Participant Center: U24 OD023176; Participant Technology Systems Center: 1 U24 OD023163; Communications and Engagement: 3 OT2 OD023205, 3 OT2 OD023206; and Community Partners: 1 OT2 OD025277, 3 OT2 OD025315, 1 OT2 OD025337, 1 OT2 OD025276. In addition, the *All of Us* Research Program would not be possible without the partnership of its wonderful participants. Research reported in this publication was supported by the National Cancer Institute of the National Institutes of Health under Award Number R15CA293800.

Author contributions

M.H.B. and M.F.B. supervised and guided data analysis. B.A.B. drafted and wrote the manuscript. M.H.B. and M.F.B. revised the manuscript. B.A.B. and M.H.B. generated the figures. K.E.B., S.A.B., D.S., and J.B. provided analysis and scientific input to *All of Us cloud* computing. C.W. contributed to manuscript editing.

Declaration of interests

The authors declare no competing interests.

Declaration of generative AI and AI-assisted technologies in the writing process

During the preparation of this work, the authors used ChatGPT to assist in structuring the abstract. After using ChatGPT, the authors reviewed and edited the content as needed. We, the authors, take full responsibility for the content of the publication.

Supplemental information

Supplemental information can be found online at https://doi.org/10.1016/j.xhgg.2025.100405.

Web resources

All analyses were performed on the *All of Us* workbench, which utilized Google Cloud Infrastructure. (https://www.researchallofus.org/data-tools/workbench/).

The majority of quality control measures were performed by the *All of Us* Research Program. https://www.researchallofus.org/wp-content/themes/research-hub-wordpress-theme/media/2022/06/All%20Of%20Us%20Q2%202022%20Release%20Genomic%20Quality%20Report.pdf.

Received: August 13, 2024
Accepted: January 8, 2025

References

1. Ellrott, K., Bailey, M.H., Saksena, G., Covington, K.R., Kandoth, C., Stewart, C., Hess, J., Ma, S., Chiotti, K.E., McLellan, M., et al. (2018). Scalable Open Science Approach for Mutation Calling of Tumor Exomes Using Multiple Genomic Pipelines. Cell Syst. *6*, 271–281.e7.
2. Bailey, M.H., Tokheim, C., Porta-Pardo, E., Sengupta, S., Bertrand, D., Weerasinghe, A., Colaprico, A., Wendl, M.C., Kim, J., Reardon, B., et al. (2018). Comprehensive Characterization of Cancer Driver Genes and Mutations. Cell *173*, 371–385.e18.
3. Hoadley, K.A., Yau, C., Hinoue, T., Wolf, D.M., Lazar, A.J., Drill, E., Shen, R., Taylor, A.M., Cherniack, A.D., Thorsson, V., et al. (2018). Cell-of-Origin Patterns Dominate the Molecular Classification of 10,000 Tumors from 33 Types of Cancer. Cell *173*, 291–304.e6.
4. Steele, C.D., Abbasi, A., Islam, S.M.A., Bowes, A.L., Khandekar, A., Haase, K., Hames-Fathi, S., Ajayi, D., Verfaillie, A., Dhami, P., et al. (2022). Signatures of copy number alterations in human cancer. Nature *606*, 984–991.
5. Taylor, A.M., Shih, J., Ha, G., Gao, G.F., Zhang, X., Berger, A.C., Schumacher, S.E., Wang, C., Hu, H., Liu, J., et al. (2018). Genomic and Functional Approaches to Understanding Cancer Aneuploidy. Cancer Cell *33*, 676–689.e3.
6. Liu, J., Lichtenberg, T., Hoadley, K.A., Poisson, L.M., Lazar, A.J., Cherniack, A.D., Kovatich, A.J., Benz, C.C., Levine, D.A., Lee, A.V., et al. (2018). An Integrated TCGA Pan-Cancer Clinical Data Resource to Drive High-Quality Survival Outcome Analytics. Cell *173*, 400–416.e11.
7. Huang, K.-L., Mashl, R.J., Wu, Y., Ritter, D.I., Wang, J., Oh, C., Paczkowska, M., Reynolds, S., Wyczalkowski, M.A., Oak, N., et al. (2018). Pathogenic Germline Variants in 10,389 Adult Cancers. Cell *173*, 355–370.e14.

8. Wang, X., Steensma, J.T., Bailey, M.H., Feng, Q., Padda, H., and Johnson, K.J. (2018). Characteristics of The Cancer Genome Atlas cases relative to U.S. general population cancer cases. Br. J. Cancer *119*, 885–892.
9. All of Us Research Program Investigators, Denny, J.C., Rutter, J.L., Goldstein, D.B., Philippakis, A., Smoller, J.W., Jenkins, G., and Dishman, E. (2019). The 'All of Us' Research Program. N. Engl. J. Med. *381*, 668–676.
10. Ahmad, A.S., Ormiston-Smith, N., and Sasieni, P.D. (2015). Trends in the lifetime risk of developing cancer in Great Britain: comparison of risk for those born from 1930 to 1960. Br. J. Cancer *112*, 943–947.
11. All of Us Research Program Genomics Investigators (2024). Genomic data in the All of Us Research Program. Nature *627*, 340–346. https://doi.org/10.1038/s41586-023-06957-x.
12. Poplin, R., Ruano-Rubio, V., DePristo, M.A., Fennell, T.J., Carneiro, M.O., Van der Auwera, G.A., Kling, D.E., Gauthier, L.D., Levy-Moonshine, A., Roazen, D., et al. (2018). Scaling accurate genetic variant discovery to tens of thousands of samples. Preprint at bioRxiv. https://doi.org/10.1101/201178.
13. Green, R.C., Berg, J.S., Grody, W.W., Kalia, S.S., Korf, B.R., Martin, C.L., McGuire, A.L., Nussbaum, R.L., O'Daniel, J.M., Ormond, K.E., et al. (2013). ACMG recommendations for reporting of incidental findings in clinical exome and genome sequencing. Genet. Med. *15*, 565–574.
14. Aschebrook-Kilfoy, B., Zakin, P., Craver, A., Shah, S., Kibriya, M.G., Stepniak, E., Ramirez, A., Clark, C., Cohn, E., Ohno-Machado, L., et al. (2022). An Overview of Cancer in the First 315,000 All of Us Participants. PLoS One *17*, e0272522.
15. Denny, J.C., Bastarache, L., Ritchie, M.D., Carroll, R.J., Zink, R., Mosley, J.D., Field, J.R., Pulley, J.M., Ramirez, A.H., Bowton, E., et al. (2013). Systematic comparison of phenome-wide association study of electronic medical record data and genome-wide association study data. Nat. Biotechnol. *31*, 1102–1110.
16. Hinrichs, A.S., Karolchik, D., Baertsch, R., Barber, G.P., Bejerano, G., Clawson, H., Diekhans, M., Furey, T.S., Harte, R.A., Hsu, F., et al. (2006). The UCSC Genome Browser Database: update 2006. Nucleic Acids Res. *34*, D590–D598.
17. Therneau, T.M., and Grambsch, P.M. (2013). Modeling Survival Data: Extending the Cox Model (Springer Science & Business Media).
18. Skol, A.D., Scott, L.J., Abecasis, G.R., and Boehnke, M. (2006). Joint analysis is more efficient than replication-based analysis for two-stage genome-wide association studies. Nat. Genet. *38*, 209–213.
19. Cook, M.B., Dawsey, S.M., Freedman, N.D., Inskip, P.D., Wichner, S.M., Quraishi, S.M., Devesa, S.S., and McGlynn, K.A. (2009). Sex disparities in cancer incidence by period and age. Cancer Epidemiol. Biomarkers Prev. *18*, 1174–1182.
20. Lawrence, M.S., Stojanov, P., Polak, P., Kryukov, G.V., Cibulskis, K., Sivachenko, A., Carter, S.L., Stewart, C., Mermel, C.H., Roberts, S.A., et al. (2013). Mutational heterogeneity in cancer and the search for new cancer-associated genes. Nature *499*, 214–218.
21. Couch, F.J., Shimelis, H., Hu, C., Hart, S.N., Polley, E.C., Na, J., Hallberg, E., Moore, R., Thomas, A., Lilyquist, J., et al. (2017). Associations Between Cancer Predisposition Testing Panel Genes and Breast Cancer. JAMA Oncol. *3*, 1190–1196.
22. Antoniou, A.C., Spurdle, A.B., Sinilnikova, O.M., Healey, S., Pooley, K.A., Schmutzler, R.K., Versmold, B., Engel, C., Meindl, A., Arnold, N., et al. (2008). Common breast cancer-predisposition alleles are associated with breast cancer risk in BRCA1 and BRCA2 mutation carriers. Am. J. Hum. Genet. *82*, 937–948.
23. Fackenthal, J.D., and Olopade, O.I. (2007). Breast cancer risk associated with BRCA1 and BRCA2 in diverse populations. Nat. Rev. Cancer *7*, 937–948.
24. Shettar, A., Damineni, S., Mukherjee, G., and Kondaiah, P. (2018). Gap junction β-2 expression is negatively associated with the estrogen receptor status in breast cancer tissues and is a regulator of breast tumorigenesis. Oncol. Rep. *40*, 3645–3653.
25. Pfeffer, F., Koczan, D., Adam, U., Benz, S., von Dobschuetz, E., Prall, F., Nizze, H., Thiesen, H.J., Hopt, U.T., and Löbler, M. (2004). Expression of connexin26 in islets of Langerhans is associated with impaired glucose tolerance in patients with pancreatic adenocarcinoma. Pancreas *29*, 284–290.
26. Denoyelle, F., Lina-Granade, G., Plauchu, H., Bruzzone, R., Chaïb, H., Lévi-Acobas, F., Weil, D., and Petit, C. (1998). Connexin 26 gene linked to a dominant deafness. Nature *393*, 319–320.
27. Spratt, D.E., Chan, T., Waldron, L., Speers, C., Feng, F.Y., Ogunwobi, O.O., and Osborne, J.R. (2016). Racial/ethnic disparities in genomic sequencing. JAMA Oncol. *2*, 1070–1074.
28. Boddu, S.P., Lin, E., Gill, V.S., Hinckley, N.B., Lai, C.H., and Renfree, K.J. (2024). Low-Income, Poor Physical Health, Poor Mental Health, and Other Social Risk Factors Are Associated With Decreased Access to Care in Patients With Carpal Tunnel Syndrome. J. Prim. Care Community Health *15*, 21501319241240348.
29. Suzuki, K., Hatzikotoulas, K., Southam, L., Taylor, H.J., Yin, X., Lorenz, K.M., Mandla, R., Huerta-Chagoya, A., Melloni, G.E.M., Kanoni, S., et al. (2024). Genetic drivers of heterogeneity in type 2 diabetes pathophysiology. Nature *627*, 347–357.
30. (2015). The future of cancer genomics. Nat. Med. *21*, 99.
31. Voelker, R. (2016). Funding the Cancer Moonshot. JAMA *315*, 1101.
32. Cancer Genome Atlas Research Network, Weinstein, J.N., Collisson, E.A., Mills, G.B., Shaw, K.R.M., Ozenberger, B.A., Ellrott, K., Shmulevich, I., Sander, C., and Stuart, J.M. (2013). The Cancer Genome Atlas Pan-Cancer analysis project. Nat. Genet. *45*, 1113–1120.
33. Slavin, T.P., Van Tongeren, L.R., Behrendt, C.E., Solomon, I., Rybak, C., Nehoray, B., Kuzmich, L., Niell-Swiller, M., Blazer, K.R., Tao, S., et al. (2018). Prospective Study of Cancer Genetic Variants: Variation in Rate of Reclassification by Ancestry. J. Natl. Cancer Inst. *110*, 1059–1066.
34. Oak, N., Cherniack, A.D., Mashl, R.J., TCGA Analysis Network, Hirsch, F.R., Ding, L., Beroukhim, R., Gümüş, Z.H., Plon, S.E., and Huang, K.L. (2020). Ancestry-specific predisposing germline variants in cancer. Genome Med. *12*, 51.
35. Wang, A., Shen, J., Rodriguez, A.A., Saunders, E.J., Chen, F., Janivara, R., Darst, B.F., Sheng, X., Xu, Y., Chou, A.J., et al. (2023). Characterizing prostate cancer risk through multi-ancestry genome-wide discovery of 187 novel risk variants. Nat. Genet. *55*, 2065–2074.
36. Park, S.L., Cheng, I., and Haiman, C.A. (2018). Genome-Wide Association Studies of Cancer in Diverse Populations. Cancer Epidemiol. Biomarkers Prev. *27*, 405–417.
37. Martin, A.R., Kanai, M., Kamatani, Y., Okada, Y., Neale, B.M., and Daly, M.J. (2019). Clinical use of current polygenic risk scores may exacerbate health disparities. Nat. Genet. *51*, 584–591.

HGGA, Volume *6*

Supplemental information

Intersection of rare pathogenic variants

from TCGA in the All of Us Research Program v6

Blaine A. Bates, Kylee E. Bates, Spencer A. Boris, Colin Wessman, David Stone, Justin Bryan, Mary F. Davis, and Matthew H. Bailey

Figure S1: Anatomical origins of neoplasms. The bar plot displays participant counts that illustrate cancer cases based on self-reported occurrences from surveys, electronic health records (EHRs), or both (according to SNOMED breakdown). Cancer events are categorized by major organ systems. If a participant had multiple cancers, they were counted in each affected organ system.

Figure S2: **Comparison of age distributions and cancer occurrence among broad groups of genetic similarities in *All of Us*. (A)** The age density distribution is presented for each ancestry, with age on the x-axis and density on the y-axis. **(B)** The mean age (x-axis) is plotted against the fraction of individuals exhibiting a cancer phenotype (y-axis) for each ancestry. Error bars represent the 95% confidence interval for the true mean age of each ancestry. **(C)** Time-to-cancer curves are created for each ancestry using the sample colors from panels A and B. Time is displayed in years on the x-axis, while the fraction of *All of Us* samples without a cancer diagnosis is shown on the y-axis. (Below) Two genetic ancestries, African (bottom-left) and European (bottom-right), indicate whether carrying rare predisposition mutations (darker shade) results in earlier cancer onset than non-carriers (lighter shade).

Figure S3: **Comparison of effect sizes between European-like and non-European-like genetic ancestries for selected *BRCA2*-cancer associations from our predisposition variant PheWAS**. Effect size is plotted on the x-axis for seven of the associations that were statistically significant in the European genetic ancestry PheWAS. Error bars show two times the standard error for the effect size.

Table S1: **Summary of TCGA germline variant data in *All of Us*.** Shows genomic position and reference/alternate alleles from TCGA as well as the genomic position and reference/alternate

alleles as specified in *All of Us*. The count of individuals in *All of Us* with a given variant is also given.

Table S2: Details for the results from the cancer-specific predisposition gene association study. Summary statistics are shown for each test that was run.

Table S3: **Details for the significant gene-phenotype associations in the pathogenic variant PheWAS (both European and non-European genetic ancestries).** Summary statistics for each association are given.

Figure S1

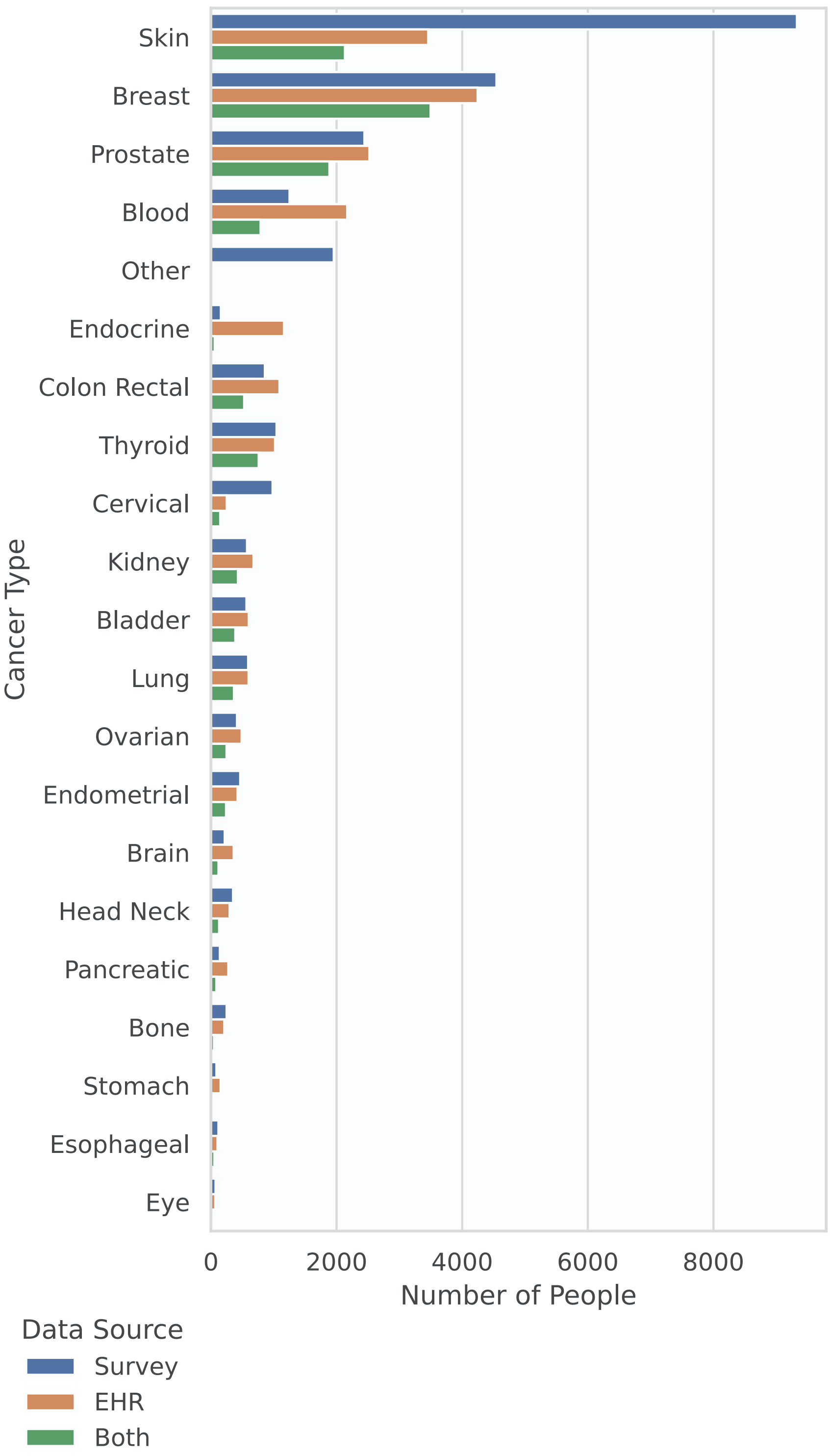

Skin
Breast
Prostate
Blood
Other
Endocrine
Colon Rectal
Thyroid
Cervical
Kidney
Bladder
Lung
Ovarian
Endometrial
Brain
Head Neck
Pancreatic
Bone
Stomach
Esophageal
Eye
Cancer Type
0
2000
4000
6000
8000
Number of People
Data Source
Survey
EHR
Both

Figure S2

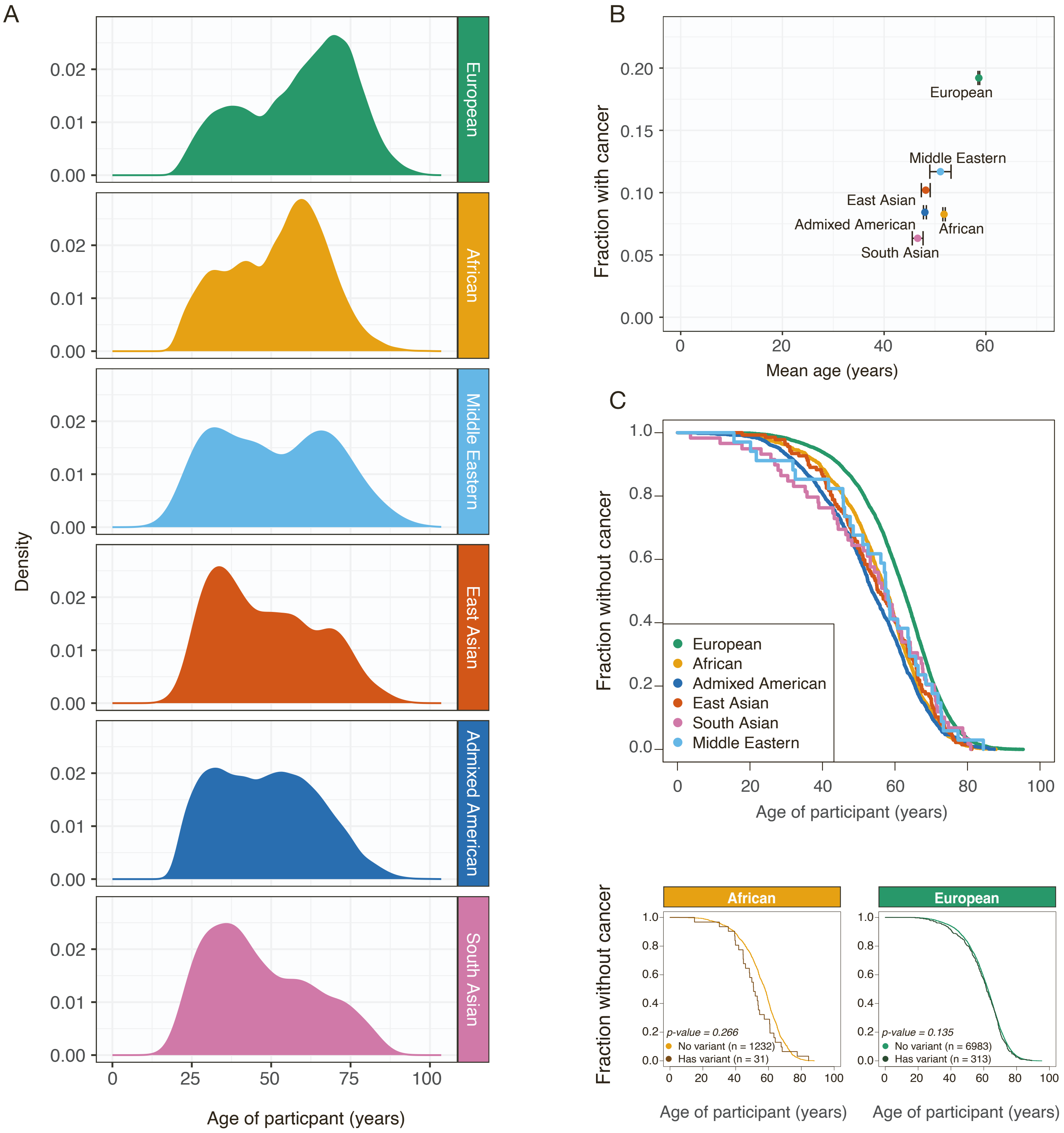
A
European
African
Middle Eastern
East Asian
Admixed American
South Asian
Density
0.00
0.01
0.02
0
25
50
75
100
Age of particpant (years)
B
Fraction with cancer
0.00
0.05
0.10
0.15
0.20
0
20
40
60
Mean age (years)
European
Middle Eastern
East Asian
Admixed American
African
South Asian
C
Fraction without cancer
0.0
0.2
0.4
0.6
0.8
1.0
0
20
40
60
80
100
Age of participant (years)
European
African
Admixed American
East Asian
South Asian
Middle Eastern
African
European
p-value = 0.266
No variant (n = 1232)
Has variant (n = 31)
p-value = 0.135
No variant (n = 6983)
Has variant (n = 313)
Fraction without cancer
Age of participant (years)
Age of participant (years)

Figure S3

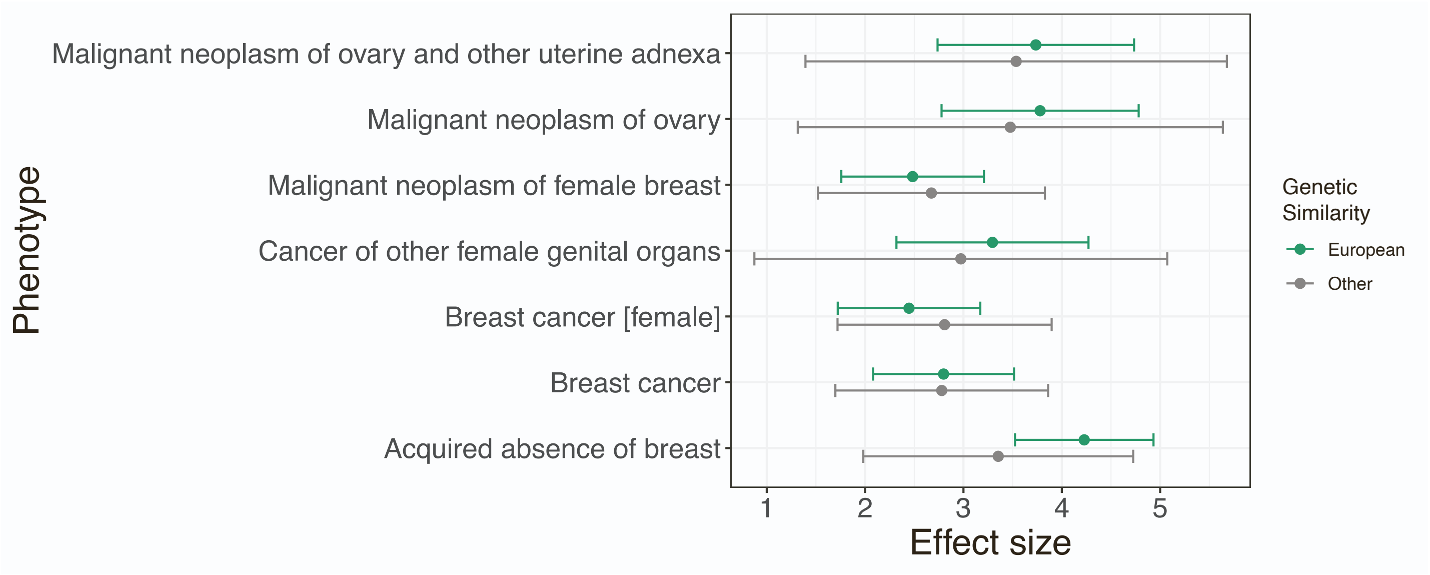
Phenotype
Malignant neoplasm of ovary and other uterine adnexa
Malignant neoplasm of ovary
Malignant neoplasm of female breast
Cancer of other female genital organs
Breast cancer [female]
Breast cancer
Acquired absence of breast
1
2
3
4
5
Effect size
Genetic Similarity
European
Other
